# Giant cell myocarditis: from immune pathogenesis to contemporary management

**DOI:** 10.1007/s10741-026-10668-6

**Published:** 2026-08-21

**Authors:** Ivan Vosko, Markus Wallner

**Affiliations:** Division of Cardiology, Department of Internal Medicine, University Heart Center Graz Medical University Graz, Auenbruggerplatz 15, Graz, 8036 Austria

**Keywords:** Giant cell myocarditis, Inflammation, Inflammatory cardiomyopathies, Endomyocardial biopsy, Immunosuppression, Cardiogenic shock

## Abstract

Giant cell myocarditis (GCM) is a rare but devastating inflammatory cardiac disease characterized by rapid hemodynamic deterioration, malignant arrhythmias, and high rates of death or heart transplantation. Without immunosuppressive treatment, median transplant-free survival is approximately three months. Current evidence identifies GCM as a predominantly T cell–mediated autoimmune disorder driven by a breakdown of immune tolerance, with contributions from macrophage-derived multinucleated giant cells and neutrophil extracellular trap formation. Viral infections and environmental factors have been implicated as potential triggers. GCM typically affects middle-aged adults, with concomitant non-cardiac autoimmune diseases present in approximately 20% of cases. Early diagnosis remains challenging, as clinical presentation and imaging findings are nonspecific and overlap considerably with cardiac sarcoidosis. Endomyocardial biopsy continues to represent the diagnostic gold standard, with a sensitivity of 85%, while cardiac biomarkers including cardiac troponins and NT-proBNP provide important prognostic information. Cardiac magnetic resonance imaging and FDG-PET contribute to the diagnostic workup but cannot reliably differentiate GCM from cardiac sarcoidosis. Management requires prompt initiation of combination immunosuppressive therapy—typically corticosteroids combined with T cell–targeted agents such as cyclosporine and azathioprine or tacrolimus and mycophenolate mofetil, which has substantially improved survival in contemporary registries. Mechanical circulatory support and heart transplantation are frequently required for refractory cases. Given the high arrhythmic burden, systematic evaluation for implantable cardioverter-defibrillator placement is recommended. This review provides a comprehensive overview of the pathogenesis, diagnosis, and current management of GCM and highlights priorities for future research, including noninvasive diagnostic tools and standardized treatment protocols.

## Introduction

Giant cell myocarditis (GCM) is a rare but frequently fulminant inflammatory disease, characterized by rapid clinical deterioration, malignant arrhythmias, and high rates of death or heart transplantation. First described in 1905 by Saltykow, GCM remains the most aggressive form of myocarditis, with a median transplant-free survival of only a few months in the absence of immunosuppressive therapy [1]. Early recognition of GCM is crucial for timely treatment and outcome but continues to be challenging. Optimal treatment regimens are not standardized, and evidence is largely derived from observational studies. Recent clinical and preclinical findings have advanced our understanding of GCM and its potential treatment. In this state-of-the-art review, we provide a comprehensive overview of the contemporary understanding of GCM, including its pathogenesis, epidemiology, clinical presentation, and diagnostic evaluation. We further discuss currently recommended therapeutic strategies, areas of controversy, and highlight priorities for future research.

## Pathogenesis, epidemiology and prognosis

### Pathogenesis

GCM is considered to be an autoimmune disease; however, the exact mechanisms are still incompletely understood. Histologically, GCM is characterized by an inflammatory infiltrate composed of lymphocytes, macrophages (histiocytes), and eosinophils, accompanied by the formation of multinucleated giant cells and widespread myocyte necrosis — in the absence of well-formed granulomas.

Gene profiling of human myocardial samples of patients with GCM, cardiac sarcoidosis, active myocarditis and inflammation-free controls using real-time quantitative polymerase chain reaction (RT-qPCR) showed that inflammatory myocardial diseases with giant cells have distinct gene profiles with elevation of certain genes coding for cytokines, cellular receptors and mitochondrial energy metabolism. In GCM samples, markers of T cell activation and infiltration were highly upregulated in comparison to the other groups, underlining the pathophysiological impact of T cells in GCM. Even though these results are in line with the general notion that GCM is an autoimmune disease, in GCM samples, Toll-like receptor 8 (TLR8) was increased. TLR8 is known to be a receptor that responds to viral RNA and may therefore link GCM to a viral trigger [2]. This is in line with various case reports of GCM after viral infections with human herpesvirus, coxsackie B2, parvovirus B19 [3] as well as SARS-CoV-2 [4].

Modern high-throughput methods allow us to deepen our understanding of GCM. Bulk RNA-Sequencing of myocardial samples of patients with GCM, lymphocytic myocarditis, acute cellular rejection in heart transplant recipients, and healthy unused donor hearts revealed a unique transcriptomic profile in GCM and 5297 differentially regulated genes. Interestingly, compared to all other groups, immune pathways of both adaptive and innate immunity were highly upregulated in GCM, while genes responsible for cardiac conduction and contraction were strongly downregulated [5]. 

It has been shown that mice with Forkhead-Box-Protein P3 (Foxp3^+^) regulatory T-cells (Tregs) depletion developed severe autoimmune-mediated myocarditis, with a histologically close resemblance to human GCM, including massive infiltration of CD4^+^ T-lymphocytes, macrophages and formation of multinucleated giant cells. Furthermore, the authors observed that these mice developed specific antibodies against cardiac myosin. However, when transferring the sera to other mice, myocarditis did not occur, suggesting a cell-mediated autoimmunity. Therefore, the proposed mechanism of GCM of this study was that the underlying defect in GCM is a defect or reduction in Foxp3^+^ Tregs, which leads to the activation of autoreactive T-cells, a CD4^+^ T-cell mediated inflammation and macrophage infiltration with a subsequent formation of giant cells and myocardial destruction. However, in this model, the Treg depletion led to other simultaneous autoimmune disorders, which is less common in humans (affecting around 20% of GCM patients) [6]. 

In a rat model of GCM, where the phenotype was acquired via direct immunization against cardiac myosin, single-cell RNA sequencing of the myocardium revealed that the immune cell composition was, as expected, strikingly different from a healthy control with two different macrophage clusters and two T-cell clusters as well as a neutrophil cluster being the most abundant immune cell subpopulations found in the GCM group. The authors found that macrophages were the major immune cell population making up the giant cells. Interestingly, the predominantly found macrophage subpopulations could not be characterized in the classic M1/M2 polarization but showed distinct features with the marker genes in the first, highly abundant cluster 1 being *Prdx1* and *Prdx5*, which are linked to autoimmunity and *Arg1* and *Ass1* that play a role in arginine homeostasis. This cluster was shown to be monocyte-derived. The second most abundant macrophage population, cluster 2, a macrophage subpopulation that had signatures of resident macrophages but also of monocyte-derived macrophages, was defined by expression of proteins of the *MS4A* family that are thought to play a role in macrophage activation and cell signaling and therefore might be associated with the formation of giant cells. Characterization of lymphocytes in this model at the single-cell level showed that T helper (Th) cells, more specifically Th17 cells, were the predominant lymphocyte subset in GCM. These cells are known to mediate inflammation through the production of cytokines such as interleukin (IL)-17, IL-21, and IL-22, and have been associated with several autoimmune disorders. Interestingly, neutrophils were amongst the top cell populations in this study in GCM samples, accounting for around a third of the total immune cells. Further investigations showed that in the most abundant neutrophil cluster, pathways that play a role in NETosis, a process that refers to the release of neutrophil extracellular traps (NETs) upon cell death of neutrophils and can cause further inflammation, were upregulated in GCM and were thought to play a role in the pathogenesis of the disease. Mechanistically, inhibition of Peptidyl arginine deiminase 4 (PAD4), an enzyme needed for NET production, led to a significant reduction in markers of NETosis, neutrophils, and indicators of inflammation in the myocardium [7]. 

**Fig. 1:**
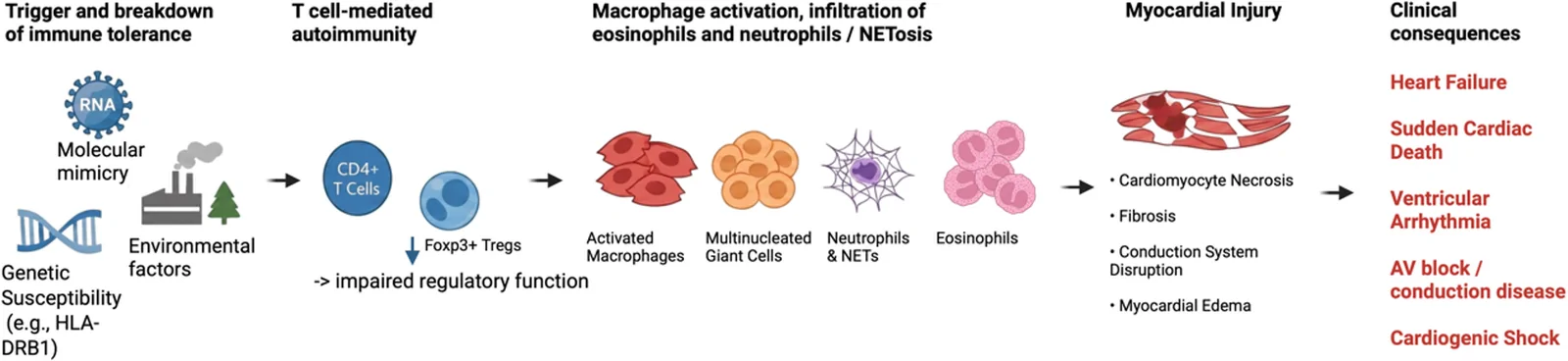
Current view on the pathogenesis of giant cell myocarditis. HLA = human leukocyte antigen, CD = Cluster of differentiation, Foxp3 = Forkhead-Box-Protein P3, Tregs = regulatory T-cells, NETs = Neutrophil extracellular traps, AV = atrioventricular. Created with BioRender.com

Taken together, as depicted in (Fig. 1), current evidence supports a model in which GCM represents a predominantly T cell–mediated autoimmune disorder driven by a breakdown of immune tolerance, potentially triggered by viral or environmental factors. This results in the activation of autoreactive CD4^+^ T cells, recruitment and activation of macrophages, and subsequent formation of multinucleated giant cells. The contribution of neutrophil extracellular trap formation is believed to further increase myocardial inflammation and promote tissue injury.

## Epidemiology

Due to its rarity and often rapidly fatal course, reliable data on true incidence are scarce. In autopsy registries, an incidence of 0.007% to 0.051% has been reported [8, 9]. A more recent study reported a frequency of 0.2% for GCM in patients undergoing EMB for suspected myocarditis or idiopathic dilated cardiomyopathy [2]. Contemporary registries show that the mean age at onset ranges from 43 to 52 years [10, 11] with GCM affecting both sexes equally and occurring in otherwise healthy individuals [10]. However, in a relatively high proportion of GCM patients, other non-cardiac autoimmune diseases were present. This finding is consistent across multiple studies, and a frequency of 17 to 19% was reported in three separate registries [10–12]. Other typical cardiovascular risk factors are less common than in other cardiovascular diseases (hypertension 29%, diabetes 5%, previous cardiovascular disease 5%) [10].

## Prognosis

Untreated, GCM is a rapidly progressive and, in most cases, lethal disease. Studies have shown that the median transplant-free survival without immunosuppressive therapy was 3 months [11]. The overall transplant-free survival in this cohort was 5.5 months. More contemporary registries still show that giant cell myocarditis is a severe disease; however, the reported death rates were markedly lower than previously described, presumably because of the frequent use of immunosuppressive combination therapy. In a Finnish registry published by Ekström and colleagues, including 51 patients with histologically proven GCM between 1991 and 2016, survival over a median follow-up of 19 months was 86%. In this study, all patients received immunosuppressive therapy, 62% received an ICD, and 19 patients (37%) required cardiac transplantation [12]. Long-term follow-up data in GCM survivors are scarce. Maleszewski and colleagues analyzed a cohort of 26 patients who survived more than one year without heart transplantation. They observed that, during subsequent follow-up, 19% required a heart transplant and one patient received a left ventricular assist device (LVAD). Interestingly, in 3 patients (12%) GCM recurred up to 8 years after the initial diagnosis [13]and can also recur in the transplanted heart [14, 15].

## Recognizing giant cell myocarditis

**Fig. 2:**
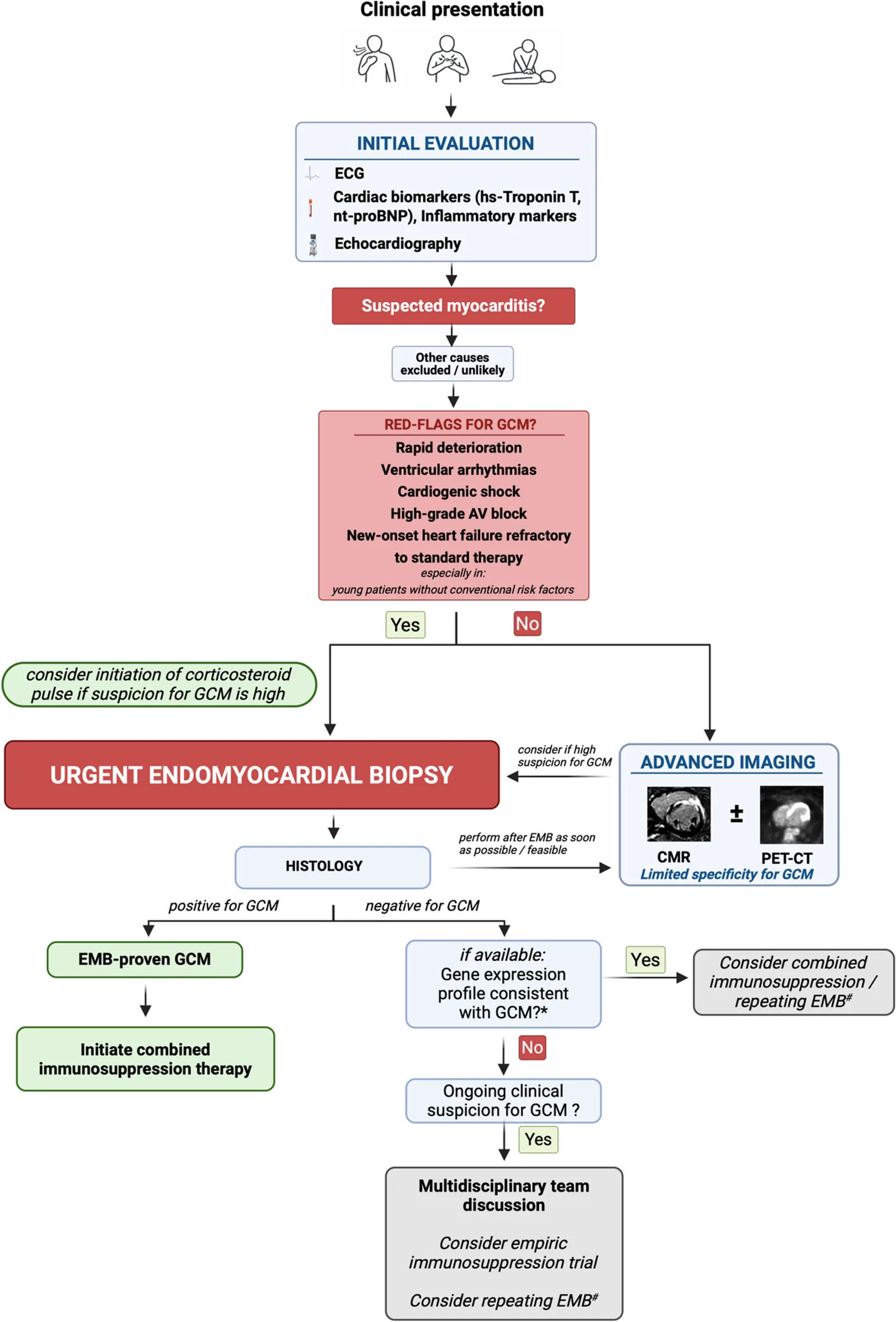
Diagnostic approach for patients with suspected giant cell myocarditis. ECG = Electrocardiogram, NT-proBNP = N-terminal pro-B-type natriuretic protein, AV = atrioventricular, CMR = cardiac magnetic resonance tomography, PET-CT = positron-emission-tomography – computed tomography, GCM = giant cell myocarditis, EMB = endomyocardial biopsy. *Gene expression profiles from EMB tissue of genes coding for cytokines and chemokines, cellular receptors, and proteins involved in the mitochondrial energy metabolism. #In case of repeated EMB consider voltage-mapping-guided EMB. Created with BioRender.com

Timely diagnosis of GCM is important but challenging. EMB remains the gold standard for diagnosis. The clinical features and diagnostic modalities are discussed in this section, and a schematic overview is provided in (Fig. 2).

## Clinical presentation

Initial symptoms are comparable to those of other types of myocarditis; therefore, GCM is often not considered initially. Most patients present with nonspecific symptoms or signs of heart failure, such as dyspnea, decreased exercise tolerance, orthopnea, and peripheral edema. Other initial cardiac symptoms include ventricular arrhythmias, high-grade AV blocks, or sudden cardiac death [10–12]. Interestingly, in a recent registry, around a third of all GCM patients were classified as New York Heart Association (NYHA) class I at presentation [12].

GCM must be considered especially in patients with suspected myocarditis and rapid clinical worsening.

### Electrocardiogram

An electrocardiogram (ECG) is an important diagnostic tool in the initial assessment. Findings on ECG in GCM include right or left bundle branch blocks, frequent premature ventricular complexes (PVCs), Q-waves, high-grade atrioventricular (AV) blocks, and ST-segment changes mimicking acute myocardial infarction [10, 16]. Malignant ventricular arrhythmias are especially frequent in patients with GCM. The rate of sustained ventricular tachycardias or ventricular fibrillation at presentation has been reported to be as high as 38% [10] and around half of all patients develop sustained ventricular arrhythmias during the subsequent course of the disease [11]. High-grade AV blocks have been reported in 9–31% of GCM cases [10, 16]. At presentation, it is important to exclude other possible causes, especially acute coronary syndromes. A normal ECG does not rule out GCM.

## Biomarkers

Several studies have demonstrated the usefulness of cardiac biomarkers in the diagnosis and risk stratification of patients with GCM. Low cardiac troponin T (< 85 ng/L) at presentation has been shown to be a good predictor of a higher probability of transplant-free survival in a cohort of 46 patients with histologically proven GCM, whereas patients with higher troponin values had markedly worse outcomes [12]. Interestingly, in a small case series, it has been reported that troponin I values at first presentation were not a reliable predictor of the diagnosis or severity of GCM [17]. 

Further, plasma levels of N-terminal pro-B-type natriuretic peptide (NT-proBNP) are typically highly elevated in GCM patients, with median values ranging from 3727 to 8309 ng/L at presentation, although normal levels have also been described and therefore do not rule out the diagnosis at presentation [10, 12]. However, higher NT-proBNP levels were shown to correlate with worse transplant-free survival; patients with an NT-proBNP > 5006 ng/L were at considerably higher risk [12]. Other biomarkers, such as markers of inflammation like high-sensitivity C-reactive protein or interleukin measurements, have not been evaluated in the context of GCM in larger cohorts to date.

## Echocardiography

Although there are no specific signs for GCM, transthoracic echocardiography (TTE) usually remains the first imaging tool used in the evaluation of patients with a clinical suspicion of giant cell myocarditis. TTE is readily available and enables good characterization of cardiac function, valve abnormalities, the presence of pericardial effusion, and fluid status. Findings on TTE are, however, nonspecific and inconsistent, and data with in-depth echocardiographic characterization in larger registries are still lacking. Echocardiographic findings in GCM range from left ventricular wall thickening, suggestive of myocardial edema, and systolic dysfunction, typically measured by left ventricular ejection fraction (LVEF), to left ventricular dilatation. At presentation, LVEF can be normal, but several studies reported that the median LVEF is typically mildly to moderately reduced [16, 18, 19]. Bobbio and colleagues found that, in comparison to cardiac sarcoidosis (CS), LVEF was significantly lower in GCM patients, with a median EF of 30% [10]. In a more recent observational study, an LVEF > 35% was associated with a favorable long-term outcome [12]. While, in acute myocarditis, global longitudinal strain was shown to be more sensitive than other echocardiographic parameters such as LVEF [20], data on GCM are still lacking.

### Cardiac magnetic resonance imaging (CMR)

CMR is a valuable tool in the detailed assessment of patients with suspected myocarditis. CMR enables detailed evaluation of ventricular function and exact tissue characterization. While the information gained might be very helpful also in GCM, in clinical practice, CMR oftentimes cannot be performed due to hemodynamic instability and frequently due to need for mechanical circulatory support. In a recent study performed by Pöyhönen and colleagues, CMRs of 18 GCM patients and 18 CS patients were evaluated and the authors found that left ventricular end-diastolic volume was significantly smaller in GCM compared to CS. Mean LVEF in GCM was at 35%, similar to echocardiographic findings. Basal septal thinning and aneurysms were more frequent in CS but could also be found in 11% of GCM patients [21]. Analysis of the localization and extent of late gadolinium enhancement (LGE) has been shown to be an important prognostic factor in several cardiac diseases, including acute myocarditis and CS [22–24].

Abnormal LGEs have been reported in nearly all GCM patients [19]. In GCM, LGE was shown to be extensive, and multifocal, affecting the right ventricular septum in most cases, as well as the anterior and inferior ventricular insertion. Other frequent sites, where LGE could be described in GCM were the RV free wall, and the anterior wall. In terms of radial distribution, subendocardial LGE as well as transmural and subepicardial LGEs were most commonly described, whereas mid-myocardial LGE was present in a smaller proportion of GCM cases [21, 25]. The so-called “hook-sign”, which describes the continuous presence of an LGE from the septum towards the RV insertion sites and RV myocardium, is an MRI sign that has been shown to be highly specific for cardiac sarcoidosis [26]. Interestingly, in GCM the “hook-sign” is present in 53%-71% of cases, with no difference observed between CS and GCM [21, 27]. In general, CS and GCM cannot be reliably differentiated by CMR. In terms of prognosis, despite the similar findings on CMR, outcomes were significantly worse in GCM patients, consistent with previous literature [21]. Presence of myocardial edema can be assessed by T2-weighted imaging. In GCM, myocardial edema, characterized by elevated T2 ratios, is frequent, ranging from 67 to 100% [21, 25]. More modern techniques such as T2 mapping, which also allow the extent of edema to be quantified, have not been systematically evaluated in the setting of GCM.

Pericardial effusions were reported in one third to one half of GCM patients [21, 27].

CMR might guide endomyocardial biopsies, especially in cases with initially negative EMBs and still high clinical suspicion.

### FDG-PET

^18^Fluorodeoxyglucose (FDG) positron-emission tomography (PET) imaging is a sensitive method in detecting areas with high metabolic activity suggestive of ongoing inflammatory processes. In GCM, the role of FDG-PET is not well established, in part because FDG-PET is often not feasible owing to hemodynamic instability. In the largest registry evaluating FDG-PET in GCM, 15 patients with histologically proven GCM underwent FDG-PET imaging, and 14 had abnormal myocardial uptake [19]. The prognostic value of FDG-PET in GCM has not been evaluated. However, FDG-PET imaging might be useful in the discrimination between CS and GCM, since also extracardiac manifestations of CS can be diagnosed. Furthermore, FDG-PET could be used to guide endomyocardial biopsy (EMB), especially in cases with high clinical suspicion and negative first EMB.

### Endomyocardial biopsy

Until 1984, the diagnosis of GCM was possible only at autopsy [11]. Since then, endomyocardial biopsy has emerged as the gold standard for the definite diagnosis of GCM, and up to now, diagnosis cannot be made without histological proof. Rapid diagnosis is of utmost importance and has a high relevance for further treatment. For patients presenting in peripheral hospitals with a high suspicion of GCM, it is recommended to transfer the patient to a center where early diagnostic EMB and further treatment options, including short-term MCS, are available. Current guidelines recommend performing early EMB in patients presenting with suspected myocarditis and high-risk features, such as acute heart failure or cardiogenic shock, marked dyspnea refractory to medical therapy, cardiac arrest, malignant ventricular arrhythmias, or unexplained high-grade AV blocks after exclusion of CAD [28].

Because GCM is frequently fulminant and the histological diagnosis is crucial for adequate therapy, endomyocardial biopsy (EMB) should be performed early rather than deferred. In the multinational FULLMOON study of 419 patients with fulminant myocarditis, compared to a delayed EMB (defined as > 2 days after admission to an ICU), performing an early EMB has been shown to be independently associated with higher transplant- or LVAD-free survival [29]. Delaying diagnosis risks the progression of a potentially treatment-responsive disease to irreversible biventricular failure. With respect to biopsy site, samples have traditionally been obtained from the right ventricle; however, selective right- or left-ventricular sampling alone yields a diagnosis in only 67% to 85% of cases. In the largest comparative series, a biventricular approach increased the diagnostic yield in patients with suspected myocarditis from 68% to 80% [30]. Therefore, a biventricular or targeted left-ventricular strategy can be considered when GCM is suspected and initial right-ventricular sampling is nondiagnostic. If the right ventricle is used, the endomyocardial biopsy should be performed from the right ventricular septum, obtaining 5 to 6 samples from different areas [9].

Histologically, GCM is characterized by extensive necrotic areas and an inflammatory infiltrate consisting of lymphocytes, macrophages, and eosinophils. The main diagnostic findings are multinucleated cells, also called giant cells. Importantly, the presence of non-caseating granulomas must be excluded, as their presence would rule out the diagnosis of GCM [11]. 

In contrast to CS, the sensitivity of EMB in GCM has been shown to be higher, at 68–85%, presumably due to a more diffuse distribution in GCM [16, 31]. 

However, a single negative or nondiagnostic biopsy does not exclude the presence of GCM. Repeated EMB has been shown to improve the sensitivity to over 90% [11, 16]. Repeat EMB should therefore be pursued when clinical suspicion remains high despite an initially negative or equivocal result. Interestingly, in some patients, heart pathology was negative after an initially diagnostic EMB for GCM. The data suggest that following immunosuppressive therapy, the diagnostic yield might decrease over time [31].

Nonetheless, it is challenging to differentiate CS from GCM, since multinucleated cells are present in both, the diagnostic yield for granulomas is very low in CS, and both diseases can present similarly. Histologically, while in GCM, necrosis and apoptosis of myocytes can be seen in the immediate vicinity of giant cells and the inflammatory infiltrate, in CS a more focal distribution has been described, with a greater extent of fibrosis and the necrotic areas being in the periphery of the inflammatory infiltrate [2]. Furthermore, in GCM, the presence of eosinophils is more common than in CS [32]. In addition, Lassner and colleagues described distinct gene profiles that were specific for GCM and CS, which can aid diagnosis in unclear cases and negative EMBs and might further help differentiate between CS and GCM beyond the histomorphological description [2]. EMB has been shown to be important not only for the diagnosis of GCM but also for prognosis. Ekström and colleagues demonstrated that the extent of myocyte necrosis and fibrosis is a strong predictor of long-term outcomes of GCM patients. They demonstrated that patients with moderate to severe myocyte necrosis were four times more likely to reach the endpoint of death or heart transplantation, and moderate or severe necrosis and fibrosis were associated with a 7-fold increase in the risk of heart transplantation and death [12]. 

### Current recommendations on treatment of GCM

#### Immunosuppression

Due to the rapid onset and potentially life-threatening course of the disease, current guidelines recommend rapid initiation of immunosuppressive treatment when the suspicion of GCM is high, even before a definite histological diagnosis [28]. The exact treatment regimens are not standardized and usually include two or more immunosuppressive agents. Recommendations must be taken with some caveats, since due to the rarity of the disease, high-quality evidence and randomized controlled trials are missing. However, data from registries show that immunosuppression is associated with better overall and transplant-free survival [11, 12]. The first cornerstone of GCM treatment is corticosteroids. In GCM, it has been suggested to use methylprednisolone at 1000 mg/d for 3 days, with subsequent reduction to prednisone at a dose of 1 mg/kg/d and further tapering at a rate of 5–10 mg/d every 6–8 weeks [33]. In a small cohort, corticosteroid monotherapy was not associated with improved survival, with a median life expectancy of less than 4 months [11]. In hemodynamically unstable patients with very high suspicion, or in patients with definite diagnosis of GCM by EMB, initiation of a combination therapy is therefore recommended. Pathophysiologically, T cells are known to play an important role in GCM. Therefore, adding a T cell – targeted agent has been established as the second cornerstone of GCM treatment. These include cyclosporine, tacrolimus, azathioprine, or mycophenolate mofetil. In an observational study from Finland, triple immunosuppressive treatment using prednisone, azathioprine, and cyclosporine was shown to be associated with numerically higher transplant-free survival (hazard ratio 0.39), although this did not reach statistical significance, possibly owing to the small number of cases [12]. Treatment with corticosteroids and cyclosporine with or without muromonab-CD3 was associated with a significant decrease in lymphocytes, giant cells, eosinophils, and necrosis in follow-up biopsies after 4 months, with no difference in LVEF [18]. Observational data evaluating the treatment regimens of 26 GCM patients from Finland showed that the transplant-free survival in patients treated with combination immunosuppressive therapy was 69% after 1 year and 58% after 2 years. Overall survival was 85% over the follow-up (median 14.5 months) [16]. Cyclosporine is mainly used in combination therapy, with a target level of 150–250 ng/mL, and the recommended dose of azathioprine is 1.5 to 2 mg/kg/day. Treatment of GCM with an immunosuppressive regimen using tacrolimus and mycophenolate mofetil combined with corticosteroids has been suggested due to superior efficacy and fewer adverse effects in solid organ transplant recipients compared with the azathioprine/cyclosporine regimen, and has been successfully used in several case reports [9, 34–36].

In fulminant or refractory cases with hemodynamic instability, the additional use of antithymocyte globulin (ATG) at a single dose of 1 mg/kg i.v., or alternatively alemtuzumab, a monoclonal antibody against CD52, at a single dose of 30 mg i.v. is recommended [33]. Applying data from bulk RNA sequencing of patients with GCM to a Drug-Gene Interaction database revealed a potential therapeutic role for other immunosuppressants, such as basiliximab, abatacept, anakinra, inotuzumab, and rituximab. However, the sample size of the derived data is very low (*n* = 4 in GCM), and it remains to be evaluated whether these drugs are also effective in actual GCM patients [5]. 

Regarding duration of therapy, the optimal length of immunosuppressive treatment remains undefined, but available evidence strongly supports prolonged therapy. In a prospective study, one patient who discontinued all immunosuppression after one year of treatment died of biopsy-proven recurrent GCM, providing direct evidence that premature cessation of immunosuppression can be fatal [18]. Furthermore, recurrence of GCM has been reported in more than 10% of survivors and as late as 8 years after the initial diagnosis [13]. After the acute phase, corticosteroids and antimetabolites (azathioprine or mycophenolate mofetil) are generally tapered after the first year. However, at least one immunosuppressant, usually a calcineurin inhibitor (cyclosporine or tacrolimus) at a low dose, is recommended to be continued for a minimum of 2 years and often indefinitely, particularly in patients with persistent left ventricular dysfunction [9]. Given the devastating consequences of disease recurrence, lifelong low-dose calcineurin inhibitor-based maintenance therapy may be a reasonable approach in many patients, although individualized risk-benefit assessment is warranted.

### Management of heart failure in GCM

Especially in stable patients with reduced left ventricular function, although specific evidence in GCM is lacking, initiation of guideline-directed medical therapy (GDMT) is generally recommended. In cases with full recovery of left ventricular systolic function, it is recommended to continue heart failure medications for at least 6 more months before slow tapering is considered [28]. 

Because GCM commonly presents as fulminant myocarditis with severe biventricular dysfunction, refractory ventricular arrhythmia, and cardiogenic shock, many patients require intensive-care management with temporary mechanical circulatory support (MCS) as a bridge to immunosuppressive response, recovery, or transplantation. In larger registries, the rate of short-term MCS (i.e., extracorporeal life support or intra-aortic balloon pump) and durable MCS (ventricular assist devices) ranged from 8% to 100%, depending largely on how recent the collected data were [37]. A French study by Montero and colleagues evaluated the use of MCS in GCM. In this registry, veno-arterial extracorporeal membrane oxygenation (VA-ECMO) was the predominant MCS used (84%), while left ventricular assist devices (LVADs) were used in 25%, all as bridge-to-transplant [37]. Use of novel microaxial flow pumps (MAFP) has not been evaluated in larger registries; however, successful use has also been described in case reports in the setting of GCM [38].

VA-ECMO is frequently the first-line device in this setting because it provides circulatory support independent of intrinsic cardiac function together with gas exchange, which is advantageous given the biventricular involvement, high arrhythmic burden, and potential for concomitant respiratory compromise commonly seen in GCM. By diverting venous return to the arterial system, VA-ECMO effectively volume-unloads the right ventricle and maintains systemic perfusion and oxygenation; it does not, however, actively unload the left ventricle. On the contrary, the retrograde aortic flow increases LV afterload, which can precipitate LV distension, pulmonary edema, and impaired myocardial recovery. In this setting, active LV unloading, for example with the additional use of a microaxial flow pump (MAFP), may be beneficial. Historically, unloading was achieved with an intra-aortic balloon pump (IABP), and much of the IABP experience in GCM derives from older cohorts, whereas contemporary practice has shifted towards the more effective unloading provided by MAFPs. MCS selection should be individualized according to the underlying physiological problem: a need for oxygenation combined with failure of systemic perfusion favors VA-ECMO, whereas predominant LV failure with preserved gas exchange may be managed with an MAFP alone or with a combined configuration (ECMELLA) when LV unloading during ECMO is required [39]. The need for heart transplantation is frequent in GCM patients. In registries, post-transplant survival was comparable to that for other underlying conditions [40]. However, GCM patients were at higher risk of acute rejection than recipients with ischemic cardiomyopathy, with no difference compared with other etiologies. Heart transplantation in GCM is most often required because of heart failure, whereas incessant ventricular arrhythmias are a less common indication [19]. Close surveillance and optimal immunosuppression are especially important in patients who received a heart transplant for GCM, because recurrences of GCM have also been described in the transplanted heart. However, in those patients the outcome of GCM was good, possibly owing to preexisting immunosuppressive therapy [11, 14].

### Management of arrhythmias

The risk of malignant ventricular arrhythmias in GCM is high, and the probability of sudden cardiac death has been reported to be 26% after 5 years of follow-up. In total, around half of all GCM patients in a larger Finnish registry were shown to experience SCD or any VT requiring treatment within 5 years [19]. In terms of medical therapy, beta blockers are the most frequently used antiarrhythmic drugs [18, 19]. This is probably due to their favorable effects in heart failure, irrespective of arrhythmic burden, and their generally good safety profile. Furthermore, amiodarone was shown to be effective in GCM, with a reduction in VT burden or complete remission in 80% of treated patients [19]. In selected patients with a high ventricular arrhythmia burden, catheter ablation can be an additional option. However, ventricular arrhythmias in GCM are frequently associated with an active and evolving inflammatory substrate, which limits the long-term efficacy of ablation and often necessitates combined antiarrhythmic and immunosuppressive management. Current evidence on the role of catheter ablation in GCM is limited to anecdotal reports [41, 42]. The acute management of refractory ventricular arrhythmia and electrical storm in GCM is necessarily extrapolated from general recommendations, as no GCM-specific comparative data exist [43]. 

Due to the reported high arrhythmic risk, every patient with known GCM should at least be evaluated for ICD implantation. In GCM, ICDs are often implanted at a low threshold; however, supporting data remain limited and recommendations rest largely on observational evidence and expert consensus. The most common indication for ICD implantation has been shown to be primary prophylaxis, whereas a smaller number of patients received an ICD for secondary prophylaxis. On long-term follow-up over 5 years, 55% of patients with an implanted ICD have been reported to receive appropriate ICD therapy [19].

**Fig. 3:**
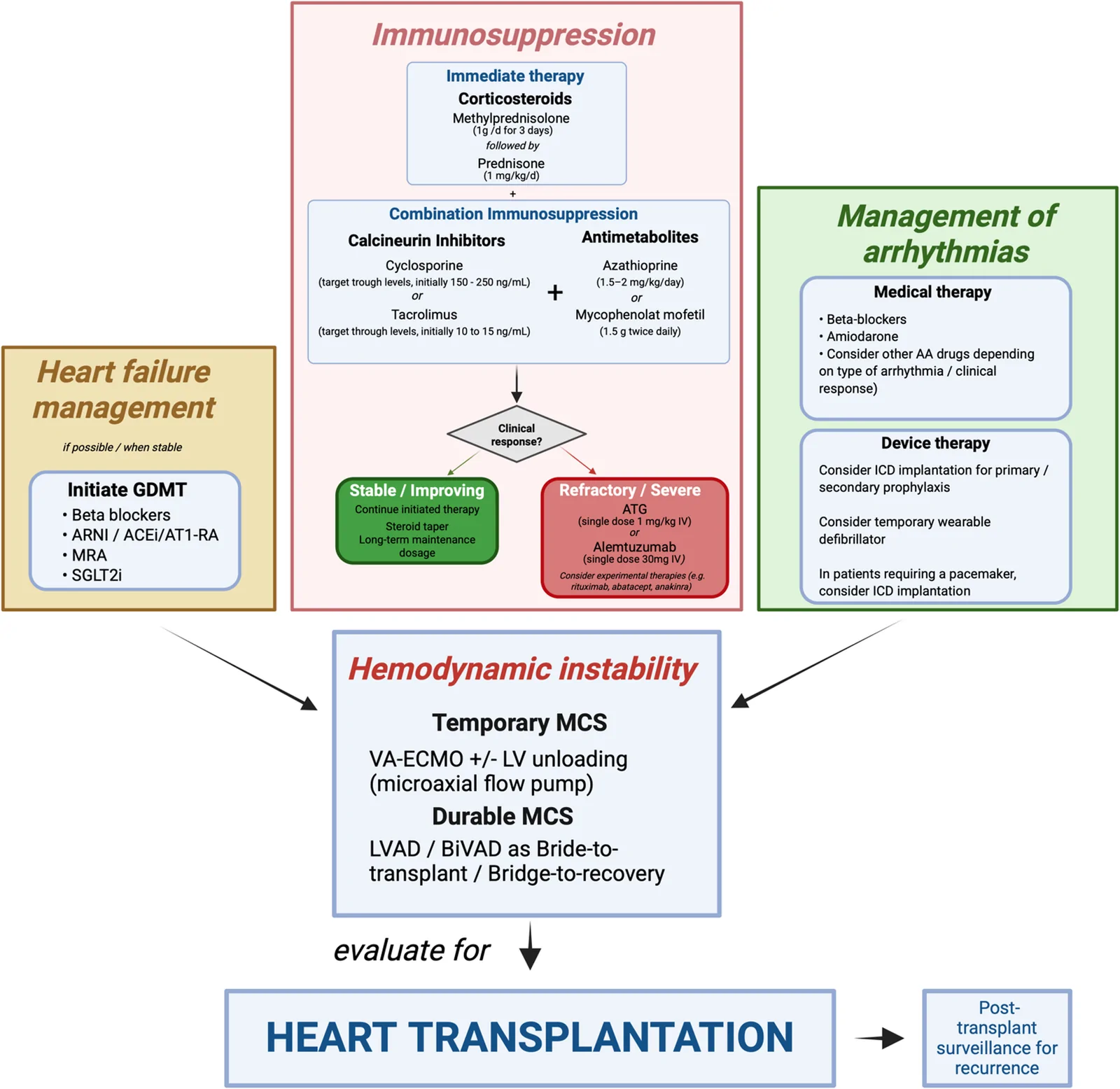
Proposed treatment algorithm for giant cell myocarditis. GDMT = guideline directed medical therapy, ARNI = angiotensin-receptor neprilysin inhibitor, ACEi = angiotensin-converting-enzyme inhibitor, AT1-RA = angiotensin 1 – receptor antagonist, Sodium glucose linked transporter 2 inhibitor, AA = antiarrhythmic, MCS = mechanical circulatory support, VA-ECMO = veno-arterial extracorporeal membrane oxygenation, LV = left ventricle, LVAD = left ventricular assist device, BiVAD = biventricular assist device. Created with Biorender.com

Interestingly, initial presentation also matters for risk stratification of arrhythmias, since it has been shown that GCM survivors who did not have ventricular arrhythmias in the initial phase of the disease were also unlikely to have arrhythmias in the long-term follow-up [13]. An overview of the currently recommended management of GCM is depicted in Fig. 3.

## Conclusion

Giant cell myocarditis is a rare but highly aggressive cardiac disease characterized by rapid progression, often biventricular failure, severe arrhythmias, and high rates of death or heart transplantation. Current evidence supports a predominantly T cell–mediated autoimmune pathogenesis, although the exact triggers remain incompletely understood.

Early diagnosis remains challenging due to nonspecific clinical and imaging findings, and therefore timely endomyocardial biopsy remains the diagnostic cornerstone. Prompt initiation of combination immunosuppressive therapy has significantly improved outcomes, as shown in predominantly observational studies; however, optimal treatment strategies are not well defined, and high-quality evidence is scarce.

Several important knowledge gaps remain. First, the optimal immunosuppressive regimen, including the choice of agents, dosing, and duration of therapy, has not been established in prospective randomized trials. Second, noninvasive biomarkers that could reliably identify GCM without the need for endomyocardial biopsy are lacking. Third, the role of novel therapeutic targets identified through transcriptomic profiling, such as NETosis inhibition or specific cytokine blockade, warrants translational investigation.

## Data Availability

No datasets were generated or analysed during the current study.
